# EVI1 as a Marker for Lymph Node Metastasis in HNSCC

**DOI:** 10.3390/ijms21030854

**Published:** 2020-01-28

**Authors:** Christian Idel, Julika Ribbat-Idel, Patrick Kuppler, Rosemarie Krupar, Anne Offermann, Wenzel Vogel, Dirk Rades, Jutta Kirfel, Barbara Wollenberg, Sven Perner

**Affiliations:** 1 Department of Otorhinolaryngology, University of Luebeck, Ratzeburger Allee 160, 23538 Luebeck, Germany; 2 Institute of Pathology, University Hospital Schleswig-Holstein, Campus Luebeck, Ratzeburger Allee 160, 23538 Luebeck, Germany; 3 Pathology, Research Center Borstel, Leibniz Lung Center, Parkallee 1-40, 23845 Borstel, Germany; rkrupar@fz-borstel.de (R.K.);; 4 Department of Radiation Oncology, University of Luebeck, Ratzeburger Allee 160, 23538 Lübeck, Germany

**Keywords:** HNSCC, EVI1, prognosis, lymph node metastasis, biomarker

## Abstract

Background: HNSCC is the sixth most common cancer in humans and has still a very poor prognosis. The treatment methods so far are very often associated with mutilation and impairment in the quality of life. Except for p16 expression, there are no reliable prognostic markers in HNSCC so far. Ecotropic Viral Integration Site 1 (EVI1) is a well-described prognostic marker in leukemia and different types of solid cancers. In these, a high EVI1 expression is associated with a poor prognosis. In HNSCC, it is not known so far if EVI1 has any prognostic relevance. Materials and Methods: We used our representative tissue cohort of 389 primary HNSCCs, of which 57.2% had one or more lymph node metastases. Here EVI1 expression was analyzed via immunohistochemistry and correlated with the clinical characteristics of these patients. Results: Although in HNSCC EVI1 expression does not predict poor survival, a high EVI1 expression in the primary tumor correlates with a lymph node metastatic disease. Conclusion: Consequently, EVI1 may serve as a biomarker to predict an occult lymph node metastasis in a clinical nodal negative (cN0) HNSCC.

## 1. Introduction

Ecotropic Viral Integration Site 1 (EVI1) was originally described in murine tumors as a retroviral integration site [1]. It is expressed in hematopoietic stem cells [2] and very well studied in myeloid leukemias as a marker for an aggressive disease with poor survival [3,4,5,6,7]. Moreover, in several solid tumors, an EVI1 overexpression indicates poor survival. Such a correlation between EVI1 overexpression and a poor outcome is described, e. g., for cancers of the colon, lung, ovary, and prostate [8,9,10,11,12,13,14]. But EVI1 is not just a prognostic marker. The relevance of EVI1 for cancer biology was demonstrated by showing that EVI1 targets some genes involved in carcinogenesis [7,15]. In sequencing analyses, HPV related uterine cervical cancer showed amplification changes for EVI1 in the 3q26 region as well [16].

Squamous cell carcinomas of the head and neck (HNSCC) are the sixth most common cancers in humans [17,18]. Typical therapies are surgery and/or radiochemotherapy, which do have rather harsh side effects. With surgical approaches, patients suffer from visible deformations and functional impairments like dysphagia or permanent voice changes. Radiochemotherapy itself leads to severe functional impairments as well. Patients suffer from dysphagia by xerostomia and necrosis, atrophy and fibrosis of the bone and different parts of soft tissue [19].

Given these therapy-induced impairments, the prognosis is still rather poor. With an increasing tumor stage, there is a decrease in survival. For UICC stage III and IV, the two year survival is around 30% as 30% to 50% develop a local or regional recurrence (LR) [18,20,21,22]. Changes in therapy regimes have not improved this fate significantly. The use of neoadjuvant and also of adjuvant chemotherapy is still controversial [20,23,24,25].

HNSCC often presents as cN0. Occult lymph node metastases (LM), however, are very frequent. So far, there is no biomarker to predict the risk of occult LM in HNSCC, but such a biomarker would be very important for the clinical decision whether treatment of the neck is necessary or not.

## 2. Results

**Figure 1:**
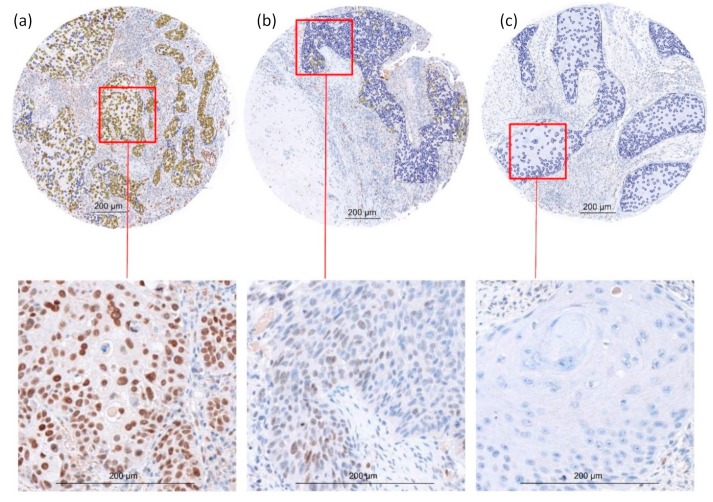
Variation of staining patterns for EVI1 in the HNSCC cohort. The tissue microarrays (TMA) were immunohistochemically stained for EVI1. Different HNSCCs showed a variation in protein expression (magnification 20×). In some HNSCCs all cancer cells showed a high homogeneous EVI1 expression (**a**). In some HNSCCs the cancer cells showed an inhomogeneous EVI1 expression with positive and negative cells (**b**), and in some HNSCCs all cancer cells showed no EVI1 expression (**c**). The lower figures show cutouts without annotated ROIs in higher magnification (40×). Scale bars represent 200 µm.

Staining of the HNSCC cohort for EVI1 showed a variety of staining patterns. In some HNSCCs, all cancer cells were highly positive for EVI1, whereas other HNSCC samples showed no EVI1 expression. Moreover, some samples showed a heterogeneous expression of EVI1 where parts of the cancer were positive while the rest was negative (Figure 1). To address these different staining patterns, the mean EVI1 expression and the positive index (ratio of EVI1 expressing cancer cells to all cancer cells within a tumor) were used for further analysis.

### 2.1. Different Sites of Origin of Primary HNSCC Have Different EVI1 Expression Levels

**Figure 2:**
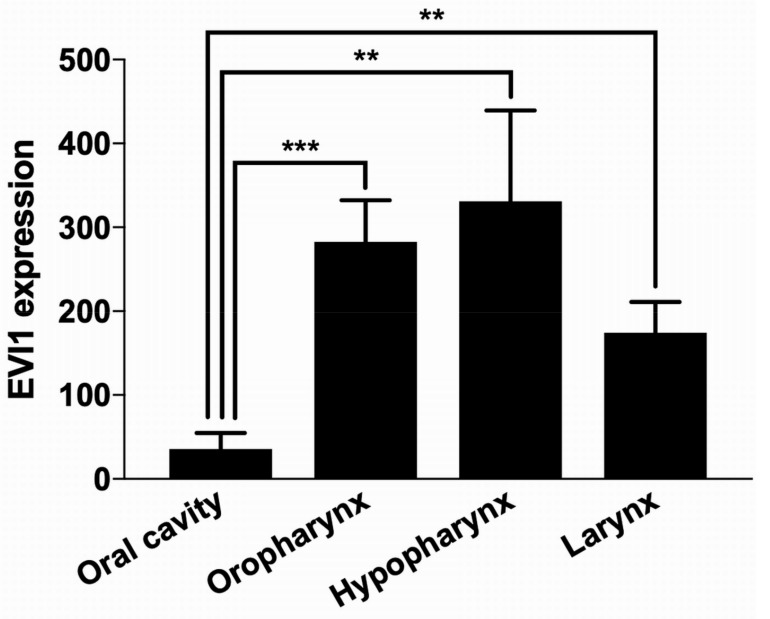
Correlation of EVI1 expression in different HNSCC sites. The primary tumors were grouped by their site of origin and the mean EVI1 expression of each group was determined. The EVI1 expression in tumors of the oral cavity is significantly lower than in other HNSCC sites (unpaired t-test, statistically significant (** *p* < 0.01; *** *p*< 0.001)).

The EVI1 expression was examined in different sites of origin of HNSCC primary tumors (PT). The highest mean expression level was detected in hypopharyngeal HNSCC (*n* = 48), followed by oropharyngeal HNSCC (*n* = 133). Laryngeal HNSCC (*n* = 111) had an intermediate mean EVI1 expression, and oral cavity HNSCC had the lowest mean expression level. The expression within the group of oral cavity HNSCC (*n* = 97) was significantly lower in comparison to the three other groups (Figure 2).

### 2.2. HPV Positive HNSCC Have Higher EVI1 Expression than HPV Negative HNSCC

**Figure 3:**
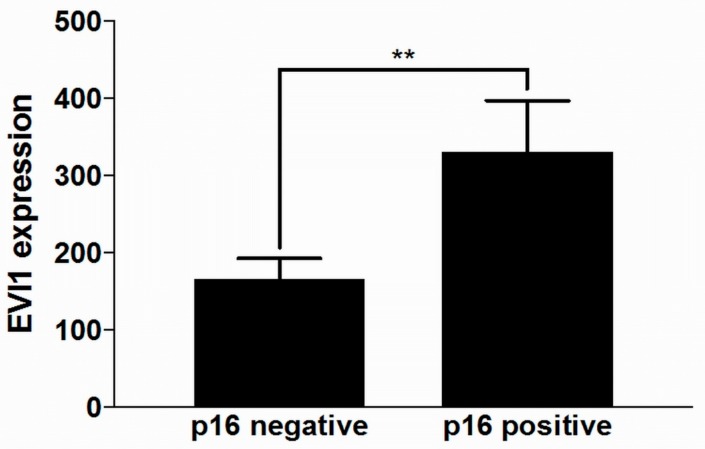
EVI1 expression in p16 positive and p16 negative HNSCC. The primary tumors were grouped by their p16 expression as p16 positive or negative. EVI1 expression in p16 positive primary tumors was significantly higher than in the p16 negative ones (unpaired t-test, statistically significant (** *p* < 0.01)).

HNSCC PTs were assessed for correlation between p16 status and EVI1. The p16 status is used as a surrogate marker for HPV infection, as recommended by the WHO and reflected in the 8th edition of the TNM classification for HNSCC [26]. Mean EVI1 expression in p16 positive tumors (*n* = 108) was significantly higher than in p16 negative tumors (*n* = 281) (*p* = 0.0054) (Figure 3).

### 2.3. Primary HNSCC with Lymph Node Metastasis Have A Higher EVI1 Expression

**Figure 4:**
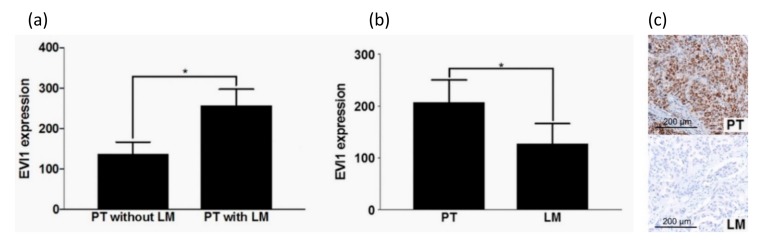
Correlation of EVI1 expression in LN+ and LN- primary HNSCC and EVI1 expression in the primary tumor and the related cervical lymph node metastasis. Primary tumors (PT) were grouped by their cervical lymph nodal status. EVI1 expression in PTs that had at least one cervical lymph node metastasis (LM) was significantly higher than in those PTs that had not formed LMs yet (**a**) (*unpaired *t*-test, statistically significant (* *p* < 0.05)). EVI1 expression of PTs, which had at least one LM, already was determined. EVI1 expression of each of these tumors was compared with EVI1 expression of the matched LM. EVI1 expression in PTs was significantly higher than in the matched LMs (**b**) (paired *t*-test, statistically significant (* *p* < 0.05)). Photomicrographs of EV11 expression of matched PT and LM are shown in (**c**). Scale bar represents 200 µm.

**Figure 5:**
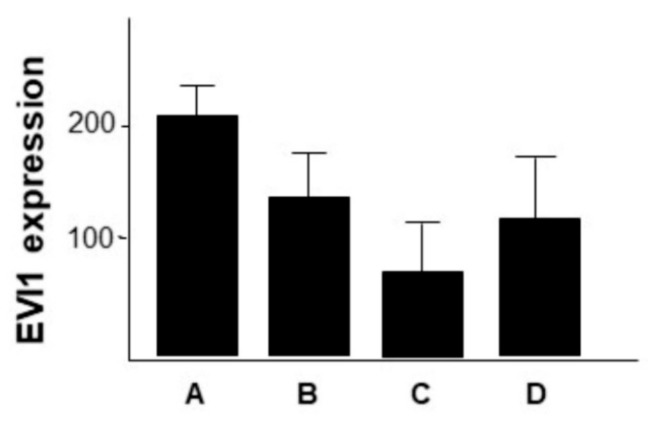
Correlation of EVI1 expression in different tumor tissues. A, primary tumor. B, (unmatched) lymph node metastasis. C, distant metastasis. D, local recurrence. The differences in EVI1 expression were not statistically significant (unpaired *t*-test, *p* > 0.05).

HNSCC PTs were assessed for correlation between EVI1 and the presence of LM. Mean EVI1 expression was significantly higher in the group of PT, which had already at least one LM at the time of first-time diagnosis (*n* = 223, *p* = 0.0212. PT without LMs = 166) (Figure 4a). Comparison of mean EVI1 expression levels between PTs (independent of nodal status, *n* = 335), LM (*n* = 169), distant metastases (DM, *n* = 25) and LRs (*n* = 69) showed there was no significant difference detectable. There was, however, a trend that the mean EVI1 expression level is highest in PTs and decreases in LMs followed by DMs and then increases again in the group of LRs (Figure 5), but in a matched pair analysis of PTs with their LMs, EVI1 expression in PTs was significantly higher than in the matching LMs (*n* = 134, *p* = 0.0456) (Figure 4b,c).

### 2.4. EVI1 Expression in HNSCC Does Not Correlate with Patient Age, UICC Stage, Overall Survival, and Disease-Free Survival

Our HNSCC cohort had a mean patient age of 62.4 years. The cohort was divided into two groups in which the EVI1 expression levels were analyzed. The first group contained PT samples of patients younger than 63 years whereas the second group consisted of all patients at the age of 63 years and older. There was no significant difference in the mean expression levels for EVI1 in these groups (see Supplemental Figure S1).

**Figure 6:**
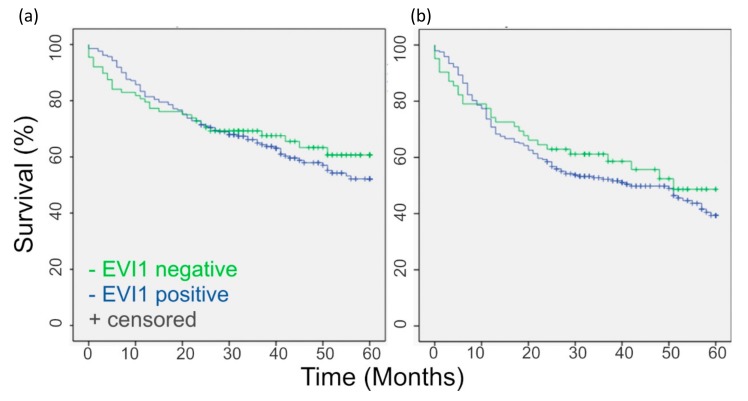
EVI1 expression does not correlate with survival data. The slight differences in five year (**a**) Overall Survival (OS) and (**b**) Disease-Free Survival (DFS) are not statistically significant (*p* > 0.05).

Afterward, PTs were assessed for correlation between UICC stages and EVI1. There was a decrease in mean EVI1 expression from stages I to III but not a statistically significant one. The mean EVI1 expression of Stage IV showed an increase in comparison to the stage III group but also not significant (see Supplemental Figure S2). There is no significant difference in overall survival and disease-free survival in EVI1 high expressing and EVI1 low expressing tumors (Figure 6).

## 3. Discussion

In HNSCC, numerous somatic copy number alterations are known [27]. One of these is located in region 3q25–3q26 which encodes EVI1 among others [28]. EVI1 has been described as an important prognostic marker in several malignant tumors. EVI1 overexpression has been shown extensively not only in hematopoietic malignancies but for several solid tumors as well. In hematopoietic malignancies, chromosomal band rearrangements of 3q26 and subsequently in the EVI1 gene are well described and lead to an EVI1 overexpression [2,29]. In ovarian cancers, a high EVI1 copy number gain was associated with a high EVI1 expression [8]. In sequencing analyses, HPV related cervical cancer showed amplification changes for EVI1 in the 3q26 region as well [16]. Yet in many cancers, the mechanisms leading to an elevated EVI1 expression are not completely known. Equally, the mechanisms leading to high EVI1 expression in HNSCC are still unknown and have not been examined so far.

The major difference between HNSCC and other solid tumor entities is that an EVI1 overexpression is not associated with a poor outcome. In the analyses presented here, p16 positive tumors showed higher EVI1 expression than p16 negative tumors. However, p16 positive tumors have a much better prognosis than p16 negative HNSCCs, a fact that was recently reflected in the 8th edition of TNM for HNSCC [30]. Furthermore, PTs with LMs showed higher EVI1 expression in comparison to PTs, which had not formed any LMs so far. In most cancer types, metastases are associated with poor survival [31]. In HNSCC, the survival correlates more reliably with an early recurrence of the tumor, which might explain why there is no prognostic impact on the overall survival by a low EVI1 expression. Actually, p16 positive HNSCCs show LMs earlier and more frequent than p16 negative tumors, but they still have a more favorable prognosis [30,32]. This might explain why a high EVI1 expression indicates a poor survival in several tumor types and not in HNSCC. For example, in colon and ovarian cancer, EVI1 is associated with a higher metastasis rate [31,33]. A formation of several subgroups by p16 status and place of origin might show that in one or the other subgroup, a high EVI1 expression is associated with a poor prognosis. In our cohort though, these subgroups turned out to be of limited statistical significance as some contained insufficient numbers of patients (data not shown).

The fact that PTs with LMs showed a higher EVI1 expression in comparison to the PTs without LMs is in analogy with the results from colon cancer, in which a high EVI1 expression is necessary to form metastases. In vivo, cancer cells with a knockdown of EVI1 failed to metastasize [31]. This might explain as well why there is a higher expression of EVI1 in p16 positive HNSCC in comparison to p16 negative HNSCC. As mentioned earlier in this discussion, p16 positive HNSCCs develop LMs earlier and more frequently than p16 negative tumors [32]. However, more studies are needed to see if the elevated EVI1 expression is due to the higher metastasis rate or if there is a direct link between p16 and EVI1.

The significantly lower expression of EVI1 in HNSCC of the oral cavity in comparison to the ones of the pharynx and larynx might be a sign that HNSCCs are not a homogeneous group. Also, the difference of EVI1 expression between PTs and their matching LMs in HNSCC underlines the hypothesis that metastases are not plain clones of their PTs but rather evolved copies. However, the observation that HNSCCs show a worse response to new therapies (e.g., checkpoint inhibitors) might be related to the fact that PTs and their metastases differ on a molecular basis [34].

## 4. Materials and Methods

### 4.1. Patient Data and Tumor Material

The study was conducted following the Declaration of Helsinki. The protocol was approved by the Ethics Committee of the University of Lübeck (project code AZ 16-277).

We established an anonymized retrospective cohort of 389 patients suffering from HNSCC, (oral HNSCC *n* = 97, oropharyngeal HNSCC *n* = 133, hypopharyngeal HNSCC *n* = 48, laryngeal HNSCC *n* = 111; 77.1% were male patients and 22.9% female; 73.1% were p16 negative and 27.9% p16 positive; 29.1% were UICC stage I/II and 70.9% UICC stage III/IV; 57.2% had at least one and 42.8% no regional LMs; 3.9% developed DM and 96.1% did not). TNM was assessed by 7th and 8th edition of TNM classification for HNSCC [26]. Patients were diagnosed between 2012 and 2015 in the Institute of Pathology of the University Medical Center Schleswig-Holstein, Campus Lübeck. They were treated in the Department of Otorhinolaryngology. Overall survival data, patient characteristics and social histories were obtained from medical records.

Representative formalin-fixed paraffin-embedded (FFPE) tumor tissues were retrieved from the archives of the Institute of Pathology of the University Medical Center Schleswig-Holstein, Campus Lübeck. Tissue samples of PTs, LMs, DMs, and LRs were arranged to create tissue microarrays (TMAs). Representative Cores from donor blocks were placed into TMA recipient blocks using a semiautomatic tissue arrayer (Beecher Instruments, Sun Prairie, WI, USA). From approx. 10%, we also utilized normal tissue. Each TMA carried up to 54 tumor samples with up to 6 normal tissue samples, each as triplet cores of 1 mm² diameter.

### 4.2. Immunohistochemistry

Immunostaining was performed on 4 μm thick sections of FFPE specimen after deparaffinization and microwave-based antigen retrieval as previously described [35]. The IView DAB Detection Kit was used on a Ventana BenchMark (Roche, Basel, Switzerland) for the EVI1 antibody (polyclonal, anti-rabbit clone C50E12, 1:200, Cell Signaling Technologies, Danvers, MA, USA) as previously described [13].

### 4.3. Evaluation of Immunohistochemical Staining

EVI1 stained slides were scanned and digitalized using Ventana iScan HT scanner (Ventana, Tuscon, AZ, USA). For digital quantification, the semi-automated image analysis software Tissue Studio (Definiens Developer XD 2.0, Definiens Inc., Carlsbad, CA, USA) was applied. This software allows to specifically mine for staining intensities in different compartments of the cell, i.e., cell nuclei, cytoplasm, membranes or whole cells in a user-specified region of interest (ROIs). Here, tumor cell areas were manually annotated for each TMA core as ROI by a pathologist in order to exclude stromal cells and benign areas from evaluation. Within that area, a continuous spectrum of tumor cell nuclei brown staining intensity (mean brown—maximum range of readout from 0 to 40) was obtained. Further, staining thresholds (hematoxylin, DAB density) were introduced to distinguish between EVI1 expressing and not-expressing tumor cells. Then, a positive index was automatically calculated that provided a ratio of EVI1 expressing cancer cells in comparison to all cancer cells within a tumor, which was used to reflect on staining homogeneity (maximum range of readout from 0 to 80). For statistical evaluation, we analyzed 3 tissue core samples of the same patient and calculated the arithmetic mean for the respective triplets. These data were finally multiplied and transformed into an expression score that displayed both EVI1 staining intensity and homogeneity of the tumor tissue. The highest readout score was 2969, which led to a maximum range of readout from 0 to 3000. Except for slide scanning, all steps of the digital analysis, including the manual ROI selection, were performed on the same computer (Windows 7 based environment, 24‘‘ monitor, resolution 1920 × 1080). This approach was published before by our group [36].

### 4.4. Statistical Analysis

Statistical analyses were performed using Prism 8 (GraphPad Software, LCC, San Diego, CA, USA) and IBM SPSS Statistics for Windows (IBM Corp., Armonk, NY, USA). Unpaired two-tailed t-test was applied to compare EVI1 expression at different PT locations and in different UICC stages as well as to discriminate EVI1 expression in p16 positive and p16 negative PTs, in PTs with or without LM disease, and in PTs, LMs, DMs and RTs. Paired t-test was used to compare EVI1 expression in PTs and matched LMs. 60-months overall and disease-free survival were calculated by Kaplan–Meier method and log-rank test for statistical significance. *p* values less than 0.05 were considered statistically significant. Bar charts show mean as columns and standard error of the mean (SEM) as error bars.

## 5. Conclusions

In summary, we shed light on the role of EVI1 in HNSCC. At the time of the first diagnosis, EVI1 expression in PT tissue could discriminate between nodal positive and nodal negative patients. This could serve as a helpful predictive tool in patient management, especially in patients that have clinically or radiologically undetectable LMs. Future studies are needed to elucidate if EVI1 can reliably predict LMs and may even be suitable for a screening approach. Hence, EV1 may serve as a promising biomarker for HNSCC patients.

## Supplementary Materials

The following are available online at https://www.mdpi.com/1422-0067/21/3/854/s1. Figure S1—EVI1 expression and patient age; Figure S2—EVI1 expression and UICC stages.

## Abbreviations

DM	Distant Metastasis
EVI1	Ecotropic Viral Integration Site 1
FFPE	Formalin-Fixed Paraffin-Embedded
HNSCC	Head and Neck Squamous Cell Carcinoma
LM	Lymph Node Metastasis
LR	Local Recurrence
PT	Primary Tumor
ROI	Region of Interest
TMA	Tissue Microarray
