# Supplementary material for: EVI1 as a Marker for Lymph Node Metastasis in HNSCC
Source: Int J Mol Sci. 2020 Jan 28;21(3):854. doi: 10.3390/ijms21030854 (PMC7038015; doi:10.3390/ijms21030854)
Supplement: Supplementary file 1 [file ijms-21-00854-s001.zip › ijms-673691-supplementary/Figure Legends Supplementary Figures.docx]

Figure Legends Supplementary FIgures

Supplementary Figure 1

EVI1 expression and patient age.

No statistically significant differences of EVI1 expression were found when comparing patients’ ages.

Supplementary Figure 2

EVI1 expression and UICC stages.

No statistically significant differences of EVI1 expression were found when comparing UICC stages.
