# Supplementary figures and images for: EVI1 as a Marker for Lymph Node Metastasis in HNSCC
Source: Int J Mol Sci. 2020 Jan 28;21(3):854. doi: 10.3390/ijms21030854 (PMC7038015; doi:10.3390/ijms21030854)

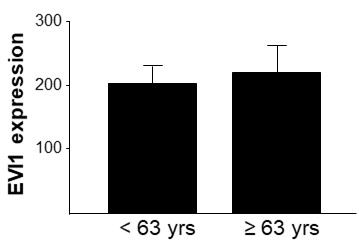

Supplement: Supplementary file 1 [file ijms-21-00854-s001.zip › ijms-673691-supplementary/supplement figure 1.jpg]

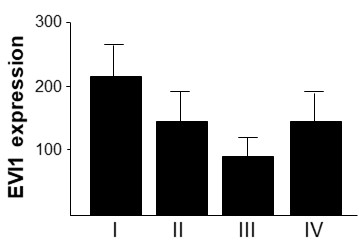

Supplement: Supplementary file 1 [file ijms-21-00854-s001.zip › ijms-673691-supplementary/supplement figure 2.JPG]
